# A Positive Feedback Mechanism That Regulates Expression of miR-9 during Neurogenesis

**DOI:** 10.1371/journal.pone.0094348

**Published:** 2014-04-08

**Authors:** Jonathan L. Davila, Loyal A. Goff, Christopher L. Ricupero, Cynthia Camarillo, Eileen N. Oni, Mavis R. Swerdel, Alana J. Toro-Ramos, Jiali Li, Ronald P. Hart

**Affiliations:** W.M. Keck Center for Collaborative Neuroscience and the Department of Cell Biology and Neuroscience, Rutgers University, Piscataway, New Jersey, United States of America; Temple University School of Medicine, United States of America

## Abstract

MiR-9, a neuron-specific miRNA, is an important regulator of neurogenesis. In this study we identify how miR-9 is regulated during early differentiation from a neural stem-like cell. We utilized two immortalized rat precursor clones, one committed to neurogenesis (L2.2) and another capable of producing both neurons and non-neuronal cells (L2.3), to reproducibly study early neurogenesis. Exogenous miR-9 is capable of increasing neurogenesis from L2.3 cells. Only one of three genomic loci capable of encoding miR-9 was regulated during neurogenesis and the promoter region of this locus contains sufficient functional elements to drive expression of a luciferase reporter in a developmentally regulated pattern. Furthermore, among a large number of potential regulatory sites encoded in this sequence, Mef2 stood out because of its known pro-neuronal role. Of four Mef2 paralogs, we found only Mef2C mRNA was regulated during neurogenesis. Removal of predicted Mef2 binding sites or knockdown of Mef2C expression reduced miR-9-2 promoter activity. Finally, the mRNA encoding the Mef2C binding partner HDAC4 was shown to be targeted by miR-9. Since HDAC4 protein could be co-immunoprecipitated with Mef2C protein or with genomic Mef2 binding sequences, we conclude that miR-9 regulation is mediated, at least in part, by Mef2C binding but that expressed miR-9 has the capacity to reduce inhibitory HDAC4, stabilizing its own expression in a positive feedback mechanism.

## Introduction

Differentiation of neural stem cells (NSCs) into neurons requires multiple transcription factors, co-activators, and co-repressors working in a coordinated, regulated manner. Large-scale gene expression analyses have been used to identify putative transcription factors and co-factors [1]–[4]. One class of co-factor includes small non-coding RNAs such as microRNAs (miRNA) and snoRNAs, which play key regulatory roles in many cellular processes, including neurogenesis, through post-translational modulation and epigenetic control [5]–[9]. Long noncoding RNAs are also essential for normal brain development [10]. MiRNAs regulate multiple cellular processes including embryonic stem cell (ESC) self-renewal [11] and neural differentiation [12]–[17]. Previous work identifying novel miRNAs and their expression profiles has established a distinct subset of miRNAs with enriched or specific expression in neural tissues and neural precursors [18]–[20]. Brain-enriched miRNAs such as miR-9, miR-124a, miR-125, and numerous others are induced in primary neural tissues and differentiating primary neurons [20]–[22]. Conversely, several ESC specific miRNAs are down-regulated during retinoic acid-induced differentiation of neuronal precursor cells [12] consistent with the hypothesis that miRNAs are likely to be key regulators of neural differentiation.

One miRNA with the potential to contribute to differentiation from a NSC to a mature neuron is miR-9 [23]. MiR-9 is expressed in proliferating and differentiating neural cells [24], [25]. Overexpression generally promotes differentiation into neurons and reduces proliferation of precursor cells [26]–[29]. It is highly conserved across species and shows CNS regional specificity in its expression [24], [30]. These findings support a key role for miR-9 during neurogenesis. However, less is known about the factors that regulate miR-9 transcriptional activity. One potential regulator of miR-9 transcription is Mef2C. Originally identified in differentiating myocytes [31], the Mef2 family of genes comprises a group of DNA-binding transcription factors belonging to the minichromosome maintenance 1-agamous-deficiens-serum response factor (MADS) family. Members of this family contain the highly conserved N-terminal MADS domain which mediates dimerization and binding activity to the A/T rich consensus sequence CTA(A/T)_4_TAG/A [32]. Gene duplications have resulted in four Mef2 paralogs (Mef2A-D) in higher vertebrates, presumably diverged from a single ancestral form, as found in organisms such as *C. elegans* and *D. melanogaster*. Extensive post-translational modification sites are strongly conserved both across lineages as well as between various family members. Adding to the complexity of the Mef2 family tree, multiple splice variants have been identified for many of the paralogs [31], [33]. Despite being initially described as exclusive to differentiating muscle, members of the Mef2 family of transcription factors have been shown to be expressed in the developing brain, and have also been shown to play crucial roles in programming early neuronal differentiation and proper distribution within the layers of the neocortex [34]. In one example, Cho *et al.* report that forced expression of a constitutively active MEF2C increases the generation of neurons with dopaminergic properties derived from hESC-derived neural progenitor cells (NPCs) [35].

As with many other MADS-containing genes, Mef2 proteins interact with a wide range of transcription factors and various other modifying proteins. This wide array of binding partners creates a diverse population of genes that are affected by Mef2 activity downstream. For instance, Mef2 genes actively bind and recruit class IIa histone deacetylases (HDACs) to promote heterochromatin formation as well as to repress target transcription activity [32], [36]. Mutations leading to nuclear accumulation of HDAC4 in neurons drastically alter patterns of chromatin marking and transcription of genes associated with Mef2 activity, directly demonstrating the function of Mef2-HDAC4 association [37]. The Mef2-HDAC4 complex has also been implicated in synaptic plasticity and a truncated variant of HDAC4 has been associated with mental retardation [38]. Clearly HDAC4, at least, is capable of altering Mef2 regulatory schemes and this mechanism may be available to regulate neural differentiation.

To understand how a miRNA-based mechanism participates in differentiation, the regulation of miRNA expression should be determined. In this study, we identified miR-9-2 as the primarily regulated miR-9 locus in differentiating neuronal precursors. Ectopic over-expression of a miR-9 mimic enhances the neurogenic differentiation capacity of a neural precursor cell. We also demonstrate that the promoter region for miR-9-2 contains two binding sites for Mef2 and show that specific inhibition of Mef2c decreases promoter activity of miR-9-2. Additionally, we identify that miR-9 negatively regulates HDAC4, a known repressor of Mef2c and reduction of HDAC4 by shRNA enhances the expression of miR-9. The repression of HDAC4 by miR-9 reinforces a positive feedback loop which enhances the neurogenic capacity of neural precursor cells.

## Materials and Methods

### Ethics statement

No animal work was completed during this study. Immortalized primary cells were obtained from Drs. Martin Grumet and Hedong Li [2], [39], [40].

### Cell culture and differentiation

Generation of neurogenic precursor clones (L2.2 and L2.3) from embryonic rat cortical cultures and their culturing conditions was described previously [2], [39], [40]. L2.2 or L2.3 cells were cultured on laminin-coated 35 mm dishes in DMEM/F12 serum free medium containing FGF2 (10 ng/ml). Differentiation was initiated by changing to medium lacking FGF2. Triplicate cultures were harvested at day 0 (+FGF2), and 1 or 3 days after differentiation (-FGF2).

### Quantitative RT-PCR

We used RNA previously isolated from L2.2 and L2.3 for qRT-PCR and microarray studies [1]. 1 μg of RNA from each of the three replicates of L2.2 and L2.3 at 0, 1, and 3 days was reverse-transcribed into cDNA using the First Strand cDNA Synthesis kit with SuperScript II reverse transcriptase (Life Technologies, Carlsbad CA). Template cDNA was amplified using Power SYBR Master Mix (Life Technologies) and qRT-PCR was carried out on either the AB7900HT or the AB7500 Fast System (Life Technologies). GAPDH was used to normalize the expression levels of each sample. Primers for all mRNA (Mef2 paralogs) and pre-miRNA qRT-PCR were designed using Primer Express 2.0 (Life Technologies) and are found in Table S1. In all cases, a reaction lacking reverse transcriptase was run to exclude the possibility that PCR primers detected genomic DNA and this was not observed (not shown). miRNA qRT-PCR was performed on the previously isolated RNA using TaqMan MicroRNA Assay (Life Technologies). qRT-PCR data were analyzed in either R (http://www.r-project.org) or Microsoft Excel. In some cases, qRT-PCR results for L2.2 cells were normalized to FGF-treated L2.3 cells from the same experiment to provide a robust signal as the denominator, reducing variance.

### Flow cytometry

PremiRs (Life Technologies) for miR-9 were nucleofected into L2.3 cells using Rat Neuron 96-well Nucleofector Kit (VHPG-1003) in conjunction with an amaxa 96-well shuttle system (Lonza, Cologne, Germany). Observed transfection efficiencies using the 96-well shuttle system were consistently >95% for both the L2.2 and L2.3 NSCs. At four hours after transfection, L2.3 cells were differentiated by removing FGF2 from the growth medium and allowed to differentiate for 72 hours. At 3 days, cells were fixed with 2% paraformaldehyde and analyzed for anti-βIII-tubulin (TuJ1, 1∶500, Covance) expression by flow cytometry on the FACSCalibur System (BD Biosciences, San Jose CA). Data was analyzed using BD's CellQuest Pro v. software.

### cDNA construction

The phylogeny of miR-9 was explored using the Sanger registry v9.0. A survey of the 5 kb upstream region of each of the miR-9 isoforms was conducted for known core promoter elements and putative transcription factor binding sites using high quality vertebrate position weight matrices (PWMs) from the Transfac 10.2 database [41]. As part of the Match algorithm, hits were selected to minimize the rate of both false positives and false negatives. As a further measure of stringency, results were selected as having a “core” nucleotide match of 100%, and tolerating a false negative rate of 10%. For miR-9-2 promoter cloning, primers were designed to allow for amplification and directional cloning into the pGL4.10 reporter vector (Promega, Madison WI). As a control, a 5 kb region directly upstream of the rat miR-9-1 precursor was also amplified and cloned into the pGL4.10 reporter vector. For site-directed mutagenesis, either or both Mef2 binding sites were removed using the QuikChange II site-directed mutagenesis kit (Stratagene, Agilent Technologies, Santa Clara, CA) from the miR-9-2 luciferase reporter plasmid.

Mef2C cDNA was prepared by reverse transcription of L2.2 RNA followed by PCR amplification of predicted protein-coding sequences (Pfu Turbo Hot Start PCR Master Mix, Stratagene, Agilent Technologies, Santa Clara, CA) and insertion into pSi expression plasmid vector (Life Technologies). Ten cDNA clones were prepared and sequenced (GeneWiz, N. Plainfield, NJ) to assess diversity of splice variants.

HDAC4 3′UTR sequences were constructed by PCR from multiple oligonucleotides with overlapping termini and inserted into the pMir-Glow Dual luciferase reporter plasmid (Fig. S4).

### shRNA Knockdown

Knockdown of Mef2C or HDAC4 was accomplished by infection with lentiviruses prepared from plasmids purchased from the TRC collection (Sigma). We selected the Mef2C shRNA for sequence specificity of the Mef2C isoform and this was confirmed by qRT-PCR of all four paralogs.

### Immunoprecipitations

Expression clones, transfection methods, and HDAC4 immunoprecipitation methods were all described previously [37]. For HDAC4-Mef2C co-immunoprecipitations, clones were transfected into N2A cells to produce larger quantities of cells. HDAC4 chromatin immunoprecipitation (ChIP) was performed using mouse brain tissues in parallel with ChIP assay reported previously [37], using the same controls.

### Luciferase assays

Target plasmids were cloned into either the promoterless pGL4.10 firefly luciferase reporter vector (Promega) or the 3′UTR-less pmiR-Report firefly luciferase reporter vector (Ambion). Plasmids were co-transfected with the Renilla luciferase control reporter vector pRL-SV40 (Promega) in a fixed concentration (0.5 μg) to normalize for differences in transfection efficiencies. Cells were maintained in culture for at least 24 hrs after transfection and then processed using the Dual-Luciferase Reporter Assay System (Promega) according to manufacturer's recommendations. Luciferase levels were quantitated using a 20/20n luminometer (Promega). Data were expressed as the ratio of firefly luciferase (FL) to Renilla luciferase (RL) to normalize for differences in transfection efficiencies.

### Statistical Analysis

Student's t-test (unpaired) or ANOVA with Tukey's post-hoc test was used as appropriate. Replicates were based on separate cultures. Any technical replicates were combined prior to statistical calculations.

## Results

**Figure 1:**
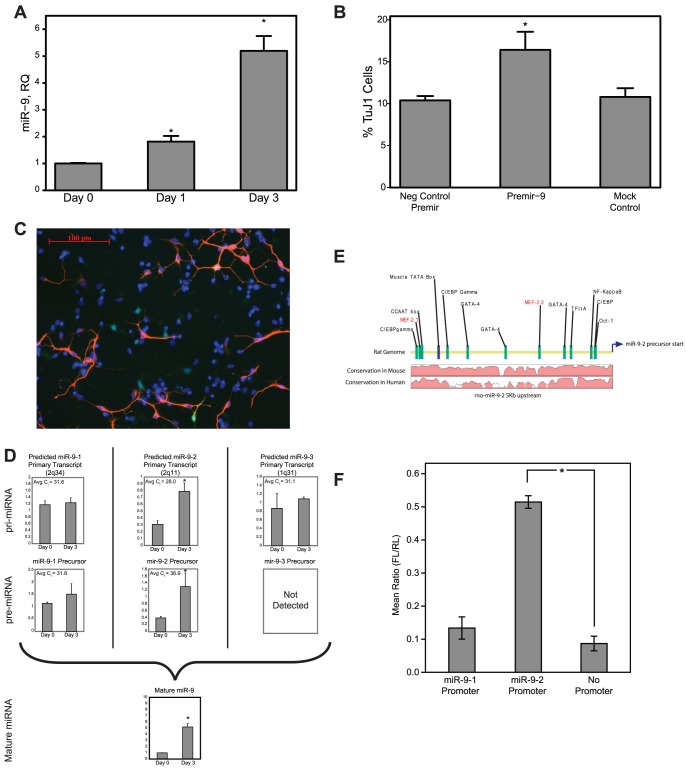
miR-9 increases during neurogenesis through transcriptional induction of the miR-9-2 locus. (A) miR-9 miRNA levels increase over time following withdrawal of bFGF from L2.2 cells. miR-9 was measured by qRT-PCR, using RNU43 as an endogenous control and results were expressed as relative quantity (RQ) compared with Day 0 control (prior to bFGF withdrawal). *p<0.05 compared with Day 0 by Tukey post-hoc test; change in miR-9 level as significant over time by ANOVA at p = 0.0003 (n = 3 per time point). (B) Ectopic addition of pre-Mir-9 mimic enhanced the proportion of TuJ1^+^ cells in multipotential L2.3 cells. TuJ1 positive cells were assayed by FACS (n = 3, *p<0.05). (C) Immunocytochemical staining of L2.3 cultures, similar to those used in Fig. 1B for FACS, showing TuJ1^+^ cells (red), non-neuronal L2.3 cells constitutively expressing GFP (green) and nuclei (blue). (D) qRT-PCR analysis of separate miR-9 genomic loci identifies miR-9-2 (2q11) as the only significantly regulated genomic locus in differentiating L2.2 cells. Data are normalized to a day 0 L2.3 standard (not plotted) to show relative differences between Day 0 and Day 3 following FGF withdrawal, but note the average cycle threshold (C_t_) values for each assay. Since the C_t_ values for the miR-9-2 products are 3–5 cycles earlier, we conclude that the transcript levels are likely 8-32-fold higher than the other alleles. (E) Transcription factor binding site predictions and sequence conservation of 5 kb upstream of miR-9-2. A 5 kb region upstream of the precursor sequence for miR-9-2 in rat was analyzed for potential transcription factor binding sites. Results shown here include two putative Mef2 binding sites, as well as a well-defined TATA box and other minimal promoter elements. Conservation plots between mouse and human demonstrate that the majority of these key TFBS occur in regions of strong conservation (>90%, see Fig. S1). (F) Luciferase reporter plasmids containing 5 kb upstream regions of two miR-9 genes demonstrate transcriptional activity of miR-9-2 after FGF withdrawal. Comparisons to the promoterless pGL4.10 were determined to be significant via Student's T-test with p<0.05. While the 5 kb region from miR-9-1 expresses a moderate amount of luciferase, the levels were not significant as compared to the promoter-less pGL4.10. Alternatively, the 5 kb region from miR-9-2 was able to significantly increase cellular levels of luciferase, indicating active transcription in L2.2 cells.

We sought to understand the mechanisms regulating miR-9 expression during neurogenesis. V-Myc-immortalized rat neural precursor cell (NPC) clones were previously used to generate a reproducible model for neural stem cell differentiation [40]. One of these clones, named L2.2, is a neuronal restricted precursor (NRP), which readily differentiates into electrically active GABAergic-like interneurons upon differentiation induced by FGF withdrawal [2]. MiRNA microarray expression profiles of L2.2 cells identified a subset of miRNAs as regulated upon differentiation. Amongst these miRNAs was miR-9. Studies have detected the expression of miR-9 in differentiating neural progenitor cells and mature mouse neurons, so we chose to focus on regulation of miR-9 in this study. To confirm the expression profile of miR-9 in differentiating L2.2 cells, we performed quantitative real-time PCR (qPCR) (Fig. 1A). MiR-9 showed a significant increase in expression upon differentiation over time following bFGF withdrawal (p<0.001, ANOVA).

If miR-9 functions in a neurogenic role during differentiation, then it should increase the percentage of neuronal cells produced during differentiation of a rat multipotential NPC clone. To test this hypothesis, we transfected miR-9 mimics into the multipotential NPC clone L2.3, which upon bFGF withdrawal produces a mix of neurons and glia [40]. At 72 hrs following electroporation of the miR-9 mimic, FACS analysis demonstrated a significant increase in the percentage of TuJ1^+^ cells (Fig. 1B & C; p<0.05, Student's t-test). While this could be due to differential cell death, we conclude that increased levels of miR-9 are sufficient to enhance neuronal pathways in uncommitted neural precursor cells.

In mammals, the mature form of miR-9 may be transcribed from one or more of three distinct genomic loci, miR-9-1, miR-9-2, or miR-9-3, likely derived from a common evolutionary ancestor gene. To explore the evolutionary relationship between these three paralogs, 78 select miRNA precursor sequences for members of the miR-9 family (File S1; miRBase v20) were aligned using ClustalW, and a phylogenetic tree was constructed from the multiple sequence alignment using the Unweighted Pair Group Method with Arithmetic Mean (UPGMA) method on the Jukes-Cantor genetic distances between aligned pairs (Fig. S1). Early vertebrate gene duplication events are evidenced by the distinct branches containing miR-9-1, miR-9-2, and miR-9-3. The lack of divergence among both the miR-9-1 and miR-9-2 isoforms indicates that these may have been the most recent duplication events, and the lower estimated per-base substitution rates for miR-9-1 and miR-9-2 family members support this claim.

Each of the miR-9 genomic loci produces a unique primary transcript that can be processed into identical, functional miR-9. It is this mature molecule which is detected by microarrays or qPCR, and therefore this assay is unable to determine the genomic origin of the mature molecule whose expression increases during neurogenesis. To identify genomic loci that are actively transcribing miR-9, we designed qPCR primers for each of the three miR-9 precursors, as well as each of three primary transcripts flanking the miR-9 precursor sequences (Fig. 1D). Results indicated that while primary transcripts were detectable for all three miR-9 genes, only the transcripts (primary and precursor) from the 2q11 region, corresponding to the miR-9-2 variant, were significantly increased during neurogenesis in L2.2 cells (p<0.05). Additionally, comparison of the average detection threshold (C_t_) values from the qPCR data suggests that the miR-9-2 primary transcript was expressed ∼8-fold higher than either of the other transcripts measured, assuming similar amplification rates. Both observations support the conclusion that only miR-9-2 is substantially transcribed, processed, and regulated during neurogenesis.

To determine whether the increase in miR-9-2 transcripts during neuronal differentiation was a product of transcriptional activation, we cloned a 5 kb genomic region upstream of the rat miR-9-2 precursor into a promoter-less luciferase expression vector (Fig. 1E). As a control, a 5 kb region upstream of miR-9-1 was also cloned. The miR-9-3 region was not cloned because we could not detect pre-miRNA from this locus in NSCs. These two plasmids, along with a negative, promoter-less control, were electroporated into neurogenic L2.2 cells along the SV40-driven Renilla luciferase plasmid as a transfection control. The ratio of Firefly luciferase to Renilla luciferase (FL/RL) showed little activity over background in the cells transfected with the miR-9-1 promoter plasmid (Fig. 1F). In contrast, increased expression of luciferase in the miR-9-2-transfected L2.2 cells suggests that this promoter is active; supporting the conclusion that miR-9-2 is the only regulated miR-9 family member during neuronal differentiation in L2.2.

To search for potential transcription factor binding sites, vertebrate position weight matrices (PWMs) from the Transfac 10.2 database [41] were utilized to scan the 5 kb region upstream of the pre-miRNAs. Interestingly, brain-specific miRNAs, including miR-9-2, are enriched for high-scoring Mef2 binding sites, as compared to a random subset of non-enriched miRNAs (Table S2). A conservation analysis of the region upstream of miR-9-2 indicates that at least two of these predicted Mef2 binding sites, denoted as Mef2.1 and Mef2.2, are in regions with a high degree of conservation between human, mouse and rat (Fig. 1F and S1C&D) suggesting a conserved role and positive selective pressure to retain functional binding sites. Several other minimal promoter elements including a TATA box and an initiator sequence were also identified upstream of miR-9-2. The Mef2 sites were selected for further study because we previously identified Mef2 binding sites to have a bioinformatically-predicted role in neural differentiation [1].

**Figure 2:**
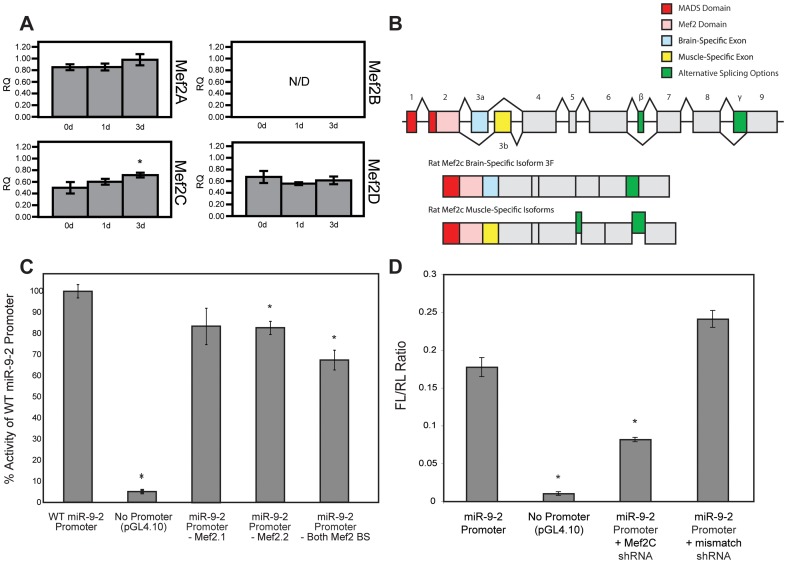
Mef2C is regulated during neurogenesis, in turn contributing to miR-9 induction. (A) qRT-PCR analysis of each of the four Mef2 paralogs during differentiation of L2.2 cells. Results are normalized to FGF-treated L2.3 cultures (not shown). Comparisons were determined to be significantly different by Student's T-test with p<0.05. As anticipated, Mef2C is the only isoform that is significantly increased during neurogenesis in the L2.2 cells. Mef2A and Mef2D transcripts both decreased during differentiation of L2.3 cells. Mef2B transcripts were not detected; however primers were able to amplify a genomic DNA positive control (not shown). (B) Splice variants of the mammalian Mef2C transcript. (C) Deletion of Mef2 binding sites in the upstream regulatory region diminishes transcription activity of the miR-9-2 gene locus. A 5 kb upstream sequence of the rat miR-9-2 gene was cloned into the promoter-less pGL4.10 luciferase reporter vector (wild-type, WT). Two highly conserved Mef2 binding sites (Mef2.1 and Mef2.2 respectively; Fig. 1E) were deleted in separate plasmids and a double-deletion plasmid was constructed as well. Plasmids were co-transfected with a Renilla transfection control reporter into undifferentiated L2.2 cultures. pGL4.10 and the full-length 5 Kb upstream sequence were transfected as controls. Ratios are expressed here as percentage of full-length miR-9-2 upstream sequence activity and differences from WT promoter were determined to be significant via Student's T-test with p<0.05. (D) Knockdown of Mef2C in neural precursors leads to a decrease in miR-9-2 promoter activity. FL/RL ratios were determined for replicate cultures of differentiated L2.2 cells with or without Mef2c shRNAs (Student's T-test, p<0.05). In this assay, a significant decrease in the abundance of luciferase was detected in the cultures electroporated with inhibitors for Mef2c. This reduction was abolished with a control hairpin with a specific mismatch for the Mef2c protein.

To examine if the different Mef2 isoforms are expressed by 3 days of neuronal differentiation of L2.2, when cultures exhibit a neuron-like morphology, we assessed the mRNA levels of Mef2 family members during neurogenesis. There was no amplification observed for Mef2B transcripts in L2.2 RNA although the primers were able to amplify purified genomic DNA as a positive control (not shown). While there was expression of each of the remaining three isoforms of Mef2, the only isoform significantly increased at 3 days after differentiation of L2.2 was Mef2C (Fig. 2A). To determine whether Mef2C was differentially spliced in NSC compared with the form previously observed in muscle [33], we prepared and sequenced cDNA clones (Fig. 2B and Fig. S2). Through direct mapping to genome, and based on homology to other conserved mammalian Mef2C genes, we propose a model of alternative splicing variants (Fig. 2B). Multiple clones were obtained for Mef2C from the L2.2 cDNA library and all were shown to contain only the brain-specific isoform 3F in each of ∼10 clones observed, suggesting this is the exclusive form of Mef2C in neural stem cells. In contrast, the optional β-exon encoding the acidic peptide N-SEDVDLLL-C was present in only ∼75% of sequenced clones. This suggests that this exon may enhance the transactivation activity of Mef2C during neural stem cell differentiation as well.

To determine whether either or both of the predicted Mef2 binding sites are required for full transcriptional activity of miR-9-2, we created deletion plasmids with either or both of the Mef2 binding sites removed. We deleted 10-12 nucleotides from the 5 kb upstream sequences of the miR-9-2 luciferase reporter plasmid corresponding to either or both of the putative Mef2 binding sites. For the Mef2.1 deletion, nucleotides between 4,779 and 4,780 upstream of the miR-9-2 transcription start site were deleted. The Mef2.2 deletion removed nucleotides 1,835 and 1,863 upstream. A third plasmid was created with both sites removed. The deletion plasmids, along with a promoter-less pGL4.10 positive control and the full-length 5 kb upstream sequence as a control, were each transfected into L2.2 cells and bFGF was withdrawn to induce differentiation (Fig. 2C). The promoter-less luciferase reporter exhibited <10% of the activity of the full-length miR-9-2 upstream region, confirming the sequence specificity of the promoter. Deletion of either the Mef2.1 or Mef2.2 binding sites resulted in a ∼20% decrease in transcriptional activity from the 5 kb upstream sequence, with only the Mef2.2 binding site demonstrating a significant decrease (p<0.05) from 100% activity. The plasmid with both conserved Mef2 binding sites removed demonstrated a 34% reduction (p<0.05 from full-length promoter), an apparently additive effect. While Mef2 is not likely to be the only mechanism governing the transcription of miR-9-2, these two Mef2 binding sites alone are capable of contributing up to one third of the transcriptional regulatory activity from the upstream region of miR-9-2 observed during differentiation of the interneuron precursor clone L2.2.

To confirm that Mef2C regulates miR-9-2 transcription, we knocked down the levels of Mef2C in differentiating L2.2 via short hairpin RNA (shRNA) targeting. We utilized commercial shRNAs that were specifically designed against discriminating regions of mouse Mef2C mRNA. Expression vectors containing shRNAs were co-transfected with the full length miR-9-2 luciferase reporter vector and a Renilla transfection control into L2.2 cells and FGF2 was subsequently withdrawn to stimulate neurogenesis. Results indicate that the levels of luciferase reporter activity are significantly reduced in the presence of Mef2c shRNA knock-down from those observed with a mismatched shRNA (Fig. 2D, p<0.05) indicating that Mef2C regulates the expression of miR-9-2. Targeting specificity was confirmed by the expression of a mouse-specific Mef2C shRNA molecule (containing a mismatch to rat) which was unable to silence Mef2C. The reduced reporter activity after knockdown of Mef2C demonstrates that this protein plays a role in regulating the transcription of miR-9-2.

**Figure 3:**
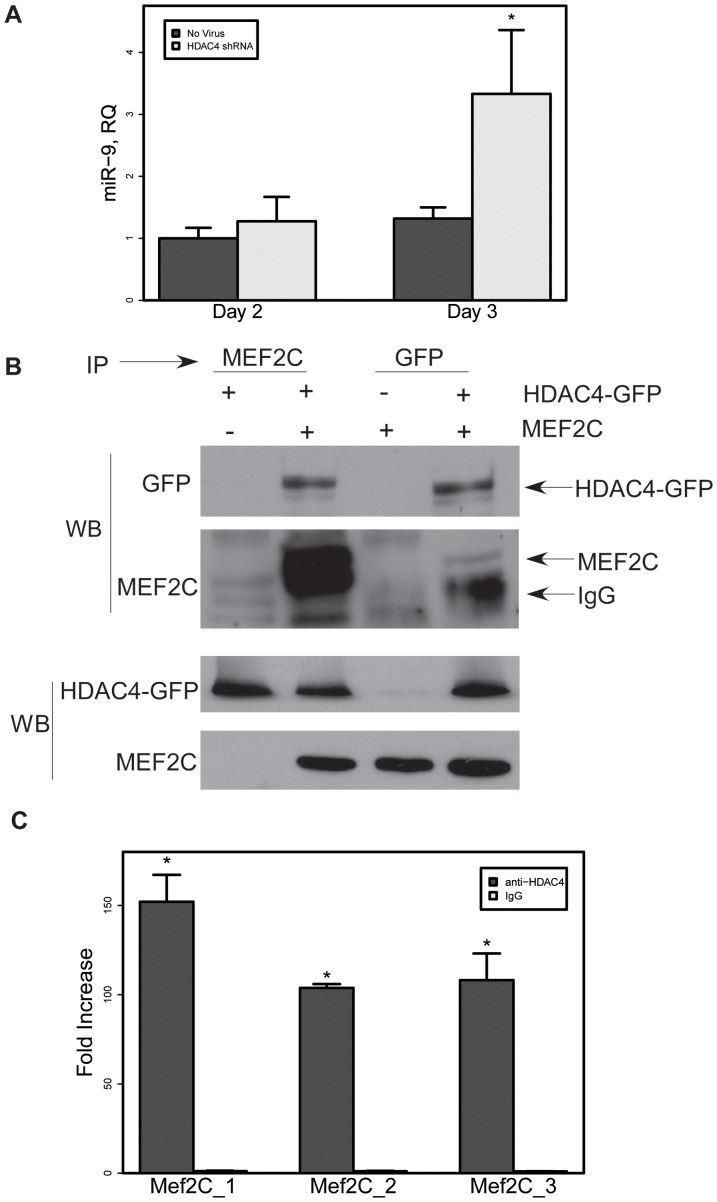
HDAC4 negatively regulates miR-9-2 promoter by binding Mef2C. (A) Infection of multipotential L2.3 cells with lentiviral-encoded shRNA specific for HDAC4 leads to increased miR-9 levels. Cultures were infected with lentiviruses and sampled 2 or 3 days later. MiR-9 was assessed using a TaqMan miRNA assay (Life Technologies). Relative Quantity (RQ) was calculated relative to RNU43 endogenous control. *p<0.05 by Student's t-test, compared with 3 days of no virus. (B) Co-immunoprecipitation of Mef2C and HDAC4. A GFP-HDAC4 fusion cDNA was expressed in N2a cells with or without a Mef2C expression clone and immunoprecipitated with antibodies against GFP or MEF2C as described previously [37]. (C) Chromatin immunoprecipitation with anti-HDAC4 enriches Mef2 binding sites upstream of mouse miR-9-2. HDAC4 immunoprecipitated chromatin from homogenized mouse brain was PCR amplified for three predicted Mef2 binding sites (Fig. S3). Primer sequences are found in Table S1. Results are expressed as fold increase relative to IgG-immunoprecipitated chromatin (*p<0.05 Student's t-test, compared with IgG).

With Mef2C acting to enhance miR-9-2 transcription, we turned to HDAC4 as a potential attenuator in this mechanism. To confirm this inhibitory role, we knocked down HDAC4 with shRNAs, expecting to see an increase in miR-9 levels due to a derepression of Mef2C. For this experiment we used the less restricted L2.3 NSC clone, hypothesizing that the HDAC4-dependent inhibitory mechanism would be more active in glial precursor cells. L2.3 cells were infected with lentiviruses encoding shRNAs against HDAC4. HDAC4 mRNA knockdown was confirmed by qPCR (not shown). MiR-9 levels were increased three days after knockdown of HDAC4 (Fig. 3A), supporting a hypothesized inhibitory role for HDAC4 on the expression of miR-9-2. Direct interaction of Mef2C and HDAC4 was confirmed by co-immunoprecipitation following transfection of both expression clones into N2a cells (Fig. 3B). Finally, since a complex of HDAC4 and Mef2C would be expected to recognize Mef2 binding sites in the genome, we confirmed that chromatin immunoprecipitation (ChIP) with HDAC4 antibody enriched three predicted Mef2 sites upstream of the miR-9-2 gene in mouse brain tissues (Fig. 3C and Fig. S3). To show specificity of the ChIP, a genomic region of the GAPDH gene was amplified and it exhibited no enrichment over IgG control in previous studies [37]. With HDAC4 shown to bind with Mef2C and to enrich the predicted Mef2 sites upstream of miR-9-2, and knockdown of HDAC4 reducing expression of miR-9, we conclude that HDAC4 attenuates miR-9 expression, likely through its interaction with Mef2C.

**Figure 4:**
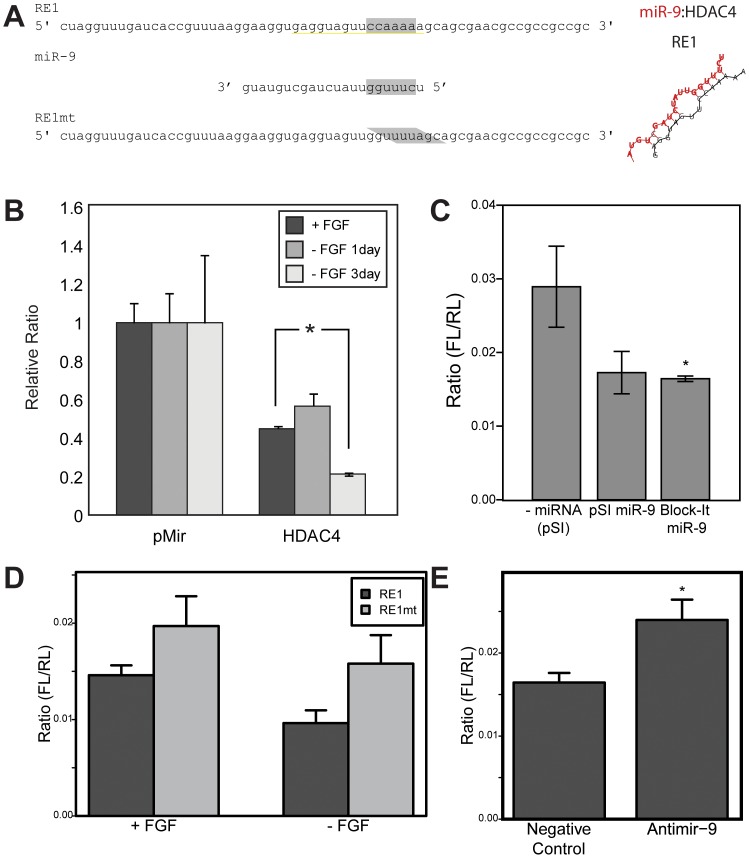
miR-9 targets HDAC4 mRNA. (A) The wild-type version of the miR-9 response element 1 (RE1) of the HDAC4 3′UTR aligned with the miR-9 sequence and the mutated form (RE1mt). (B) Differentiation of L2.2 cells causes repression of a 1.2 kb fragment of the HDAC4 3′UTR containing miR-9 response element. Results show the FL/RL luciferase ratio expressed relative to the pMir control to compare multiple differentiation days. The reduced luciferase expression at Day 3 correlates with increased miR-9 expression upon FGF withdrawal-induced differentiation. (C) Exogenous expression of miR-9 reduces luciferase activity from a clone containing the 1.2 kb fragment of the HDAC4 3′UTR containing miR-9 response element. Levels of miR-9 were increased by transfecting HeLa cells with either a plasmid vector expressing rat genomic sequences surrounding miR-9 (pSI miR-9) or a synthetic miR-9 gene constructed in the Block-IT expression vector (Life Technologies). Both cases led to increased miR-9 levels as assayed by qPCR (not shown). (D) A single predicted miR-9 wild-type (RE1) or mutant (RE1mt) response element was cloned into the pMir luciferase construct. Mutating the miR-9 RE derepresses luciferase expression, with or without FGF (p = 0.029 for RE1 vs. RE1mt, ANOVA). pMir vector containing the miR-9 RE1 was transfected into L2.2 cells. Cells were assayed for luciferase one day later, either with or without FGF. (E) Inhibiting endogenous miR-9 levels in L2.2 cells using Anti-miR-9 increases luciferase expression. Anti-miR-9 or scrambled negative control RNA (Life Technologies) were nucleofected into L2.2 cells. Cells were collected for luciferase assays after one day. *p<0.05 by Student's t-test.

Since we predict that Mef2C and miR-9 are coordinately involved in neurogenesis and HDAC4 opposes this mechanism, we searched for potential feedback networks between Mef2C and miR-9. We used TargetScanS [42], [43] to determine if any of these mRNAs were potential targets of miR-9. The 3′UTR of HDAC4 contained four predicted miR-9 response elements (RE) including one closest to the coding sequence (Fig. 4A) that is conserved with mouse and humans. A 1.2 kb fragment of HDAC4 3′UTR was cloned from L2.2 cell cDNA into the pmiR-Report luciferase expression vector. This was co-electroporated with a Renilla control plasmid into L2.2 cells. Negative control transfections of the luciferase vector without a 3′UTR were included as well. Cultures were subject to differentiation by FGF withdrawal for 0, 1, or 3 days. At each time point, cells were harvested and luciferase levels were measured. Firefly luciferase levels were corrected for transfection efficiency by normalization to Renilla expression levels (FL/RL). Furthermore, to correct for transcriptional variation as a result of FGF withdrawal, the FL/RL ratios were normalized to the FL/RL values observed for the control pmiR vector at each time point. As predicted, the normalized firefly luciferase levels were significantly decreased in the HDAC4 3′UTR plasmid expressing L2.2 cells by 3 days of differentiation (p<0.01), predicting a negative regulation of HDAC4 via 3′UTR activity during neurogenesis (Fig. 4B). To confirm that this inhibition is partially regulated by miR-9 the HDAC4 3′UTR plasmid was co-transfected with expression vectors for miR-9 (cloned into pSI and/or Block-it) into HeLa cells. HeLa cells were chosen for their inherently low background levels of neurogenic miRNAs. Data from these assays indicate that miR-9 is capable of targeting the HDAC4 3′UTR (Fig. 4C).

We further confirmed that miR-9 was directly responsible for the post-transcriptional regulation of HDAC4 by testing the activity of one of the miR-9 response elements in the HDAC4 3′UTR (Fig. 4A). The wild-type (RE1) or mutant (RE1mt) response element was cloned into the 3′UTR of firefly luciferase in the pmiR plasmid. These constructs were nucleofected into L2.2 cells and plated in presence or absence of FGF (Fig. 4D). 24 hrs after electroporation luciferase activity showed a 25% reduction in the RE1 constructs when compared to the RE1mt in cells grown in the presence of FGF, increasing to a 40% reduction when FGF was removed from the cultures (p < 0.05, ANOVA). This increase in inhibition correlates with an increase in the levels of miR-9 upon L2.2 differentiation (Fig. 1A). To confirm that the inhibition in luciferase activity seen by RE1 is caused by miR-9, the RE1 reporter plasmid was nucleofected into L2.2 cells along with either a miR-9 anti-miR (Ambion) or a negative control oligo. Cells were assayed for luciferase activity after 24 hrs. The anti miR-9 oligo was able to reverse the inhibition caused by miR-9 (*p<0.05, Fig. 4E), illustrating the specificity of miR-9 in this regulatory mechanism.

## Discussion

MiRNA regulatory networks are believed to have evolved under natural selection in order to stabilize specific phenotypes [44]. During differentiation of neural precursor cells, regulated miRNAs are likely to modulate or restrict expression of genes which can repress differentiation or are associated with alternate cellular fates. Therefore, miRNA regulation during NSC differentiation would serve to canalize neuronal phenotypes. In this study, we report that miR-9 enhances the neurogenic differentiation capacity of NPCs. Furthermore, during differentiation, Mef2C activates miR-9-2 by binding upstream regulatory sites, resulting in increased miR-9 expression. Increased miR-9 levels in turn serve to attenuate the Mef2 inhibitor HDAC4. The repression of HDAC4 by miR-9 reinforces a positive feed-back scheme that enhances the neurogenic capacity of neural precursor cells.

We show that ectopic over-expression of a miR-9 mimic enhances the neurogenic differentiation capacity of rat NPCs. In NPCs derived from mouse ESCs, miR-9 knockdown causes a reduction in the number of differentiating neurons accompanied by a slight increase in GFAP^+^ astrocytes [27]. In late embryonic zebrafish brains, miR-9 expression shows spatial specificity, avoiding expression in the midbrain-hindbrain boundary (MHB) region, a non-neurogenic boundary zone containing a pool of progenitor cells that contributes neurons to the midbrain-hindbrain domains. This spatial specificity has been proposed to be critical for regulation of FGF signaling and the maintenance of a neural progenitor state *in vivo* [45]. MiR-9 over-expression was shown to promote premature neuronal differentiation in the MHB, meanwhile, knockdown of miR-9 with modified antisense oligonucleotides (morpholino) had the opposing effect by increasing the MHB area size and region specific markers [45]. It should be noted that we did not rule out a cell-type-specific cell death following treatment with a miR-9 mimic. In another study, Zhao *et al.* showed that knock down of miR-9 in adult mouse NSCs caused a small increase in proliferating cells (1.37-fold) and that over-expression of miR-9 leads to a decrease in proliferation of precursor cells and an increase in both glial and neuronal differentiation [28]. Also, miR-9 is expressed in neural progenitor cells of *X. tropicalis*, and its knockdown results in an inhibition of neurogenesis along the anterior-posterior axis. However, the underlying mechanism differs in the hindbrain; progenitors fail to exit the cell cycle, whereas in the forebrain they undergo apoptosis, counteracting the proliferative effect [46]. However, in neural progenitor cells derived from human ESCs, *loss* of miR-9 has been shown to suppress proliferation and promote migration of neural progenitors, but has no effect on differentiation [47]. The differences among studies can partially be attributed to differences in the model systems or growth conditions, but, these discrepancies also raise the possibility that the function of miR-9 in neurogenesis and proliferation is dependent on timing and/or anatomy.

Several transcription factor pathways have been identified as targets of miR-9 during neurogenic differentiation. MiR-9 increases retinoic acid induced neuronal differentiation in neuroblastoma cells by inhibiting the neuronal differentiation repressor ID2 [26]. Gain and loss of function experiments have shown that miR-9 regulates differentiation of Cajal-Retzius cells in the medial pallium by targeting Foxg1 [48]. MiR-9 knockdown caused a reduction of Cajal-Retzius neurons but did not affect progenitor cells [48]. MiR-9 suppresses Nr2e1 (also known as TLX) expression to negatively regulate neural stem cell proliferation and accelerate neural differentiation [28] and this is likely an indirect effect of let-7d regulation [49]. MiR-9 regulates Hes1, which is expressed in an oscillatory fashion during neural progenitor proliferation but switches to increased miR-9 and less Hes1 as differentiation proceeds [29], [50]. While this list of miR-9 targets is substantial, there are likely to be many more nodes in the network of miR-9 and neurogenesis.

We identified miR-9-2 as the primarily-regulated miR-9 locus in differentiating neuronal precursors. A phylogenetic analysis of the evolutionary relationships between miR-9 genes shows that there have been several duplication events within the miR-9 family throughout the course of evolution (Fig. S1). Although the origin of the ancestral miR-9 gene cannot be determined from this analysis, it is clear that both the miR-9-2 and miR-9-3 genes arose from duplication events that also allowed for duplication of neighboring genes. We see that immediately upstream of rno-miR-9-1 and rno-miR-9-2 paralogs of the Mef2 family of transcription factors were also duplicated (Fig. S1B). Both Mef2C and Mef2D are found adjacent to separate miR-9 paralogs. The Mef2D gene is located adjacent to the miR-9-1 gene at 2q34 while the Mef2C isoform is juxtaposed next to miR-9-2 at 2q11. The limited divergence of the most recent miR-9 branch, as compared to the miR-9-1 and miR-9-3 branches, suggests that either the miR-9-2 locus is a more recent evolutionary event, or has been under greater pressure to remain unchanged. This distinction is significant here due to the singular role played by the miR-9-2 locus in increasing the cellular levels of mature miR-9 during differentiation of L2.2 cells. The duplication of this miRNA implies a positive selection mechanism to retain this activity in the mammalian brain. Also, the close genomic proximity of miR-9-2 and Mef2C, and their common pro-neuronal roles would predict that these two genes may be co-regulated at the chromatin level.

Interestingly, our results indicate that the promoter region for rat miR-9-2 contains two putative binding sites for Mef2 and that specific inhibition of Mef2C decreases promoter activity of miR-9-2. Our experiments can be interpreted to demonstrate that a inhibition of Mef2C reduces promoter activity but does not rule out that other Mef2 family members may also contribute to the activity. Cho *et al.* report that forced expression of a constitutively active Mef2c increases the generation of neurons with dopaminergic properties derived from hESC-derived NPCs [35]. These results agree with the hypothesis that the induction of miR-9-2 during neurogenesis is in part due to the activity of Mef2C, the only regulated isoform of Mef2 during neurogenesis. We sequenced cDNAs of the primary splice variant expressed from the Mef2C gene and found that they included the β exon (Fig. 2B), thought to enhance transactivation activity [33]. In addition despite the clear preference for the brain-specific exon 3 in the cDNA clones derived from L2.2, there is little known about the function of this peptide; specifically anything that may distinguish it from the activity of the muscle-specific isoform. Conserved domain scans using PantherDB, Prosite ExPASy, and CDD (NCBI) were only able to identify the conserved MADS-MEF2 domain and could not ascribe a function to any portion of this exon. Future analysis of the function of this exon may demonstrate its functional role in Mef2C induction of neurogenesis. There are many other predicted transcription factor binding sites in addition to the Mef2 binding motifs in the miR-9-2 promoter (Fig. 1E), including TLX [28], Hes1 [29], and CREB/REST [51]. These also potentially have roles in regulating the transcriptional activity of the miR-9-2 promoter and could serve to initiate miR-9 transcription in order to start the regulatory scheme. The requirement of the Mef2 transcription factor binding sites to achieve full transcription activity, combined with the dramatic effect of Mef2C knockdown on the expression of luciferase in this assay supports the hypothesis that Mef2C binding to the upstream region of miR-9-2 is capable of affecting the expression of miR-9 and suggests that Mef2C and miR-9 cooperatively interact to promote the neuronal phenotype

HDAC4 is a known repressor of Mef2 factors [36], [38], [52]–[60]. We show that HDAC4 protein is co-immunoprecipitated with Mef2C (Fig. 3B) and that anti-HDAC4 immunoprecipitates miR-9-2 promoter sequences including the Mef2 binding sites (Fig. 3C) We attempted to enrich the Mef2 sites using similar methods but we were unable to find specific enrichment of these sites or any control Mef2 sites with available antibodies (not shown). While HDAC4 is classified as a histone deacetylase, class IIa HDACs have little or no enzyme activity due to sequence variations in the active site [61] and so their inhibitory activity when bound with Mef2 is likely due to other mechanisms [38]. We also searched our own ChIP data for HDAC4 [37] and data obtained from other studies [38] and were unable to find additional HDAC4-enriched sequences near miR-9-2, but we cannot rule out that there may be other sites of interaction. We previously found that increased nuclear HDAC4 contributed to neurodegeneration in Ataxia-telangiectasia, at least in part via Mef2 [37]. We conclude that miR-9 regulation is mediated, at least in part, by Mef2C binding to the miR-9-2 promoter and that HDAC4 can serve as a repressor of MEF2C in NSCs.

**Figure 5:**
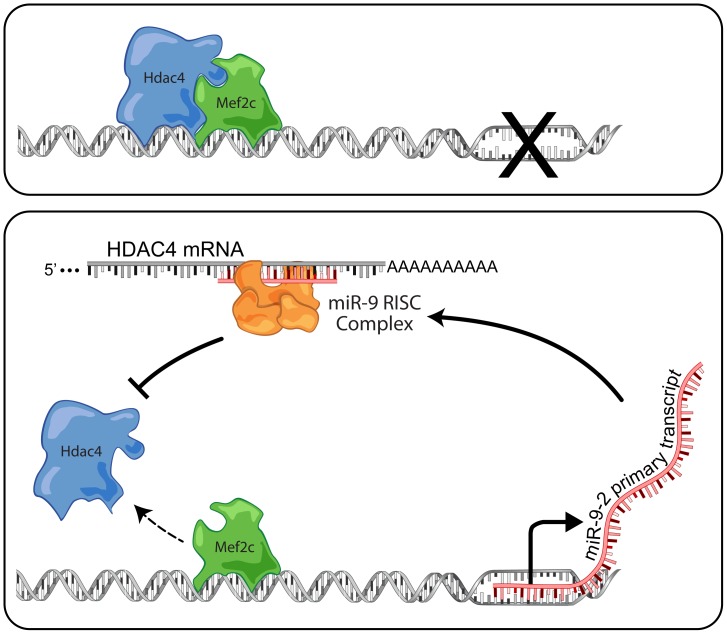
Model of Mef2c/HDAC4 regulation of miR-9 expression during neurogenesis. Cells becoming non-neuronal cells (top) would express both MEF2C and HDAC4, forming an inhibitory complex binding with sites upstream of miR-9-2 to suppress transcription. Cells becoming neurons (bottom) switch to a state where miR-9 inhibits production of HDAC4, allowing a pro-transcriptional binding of MEF2C upstream of miR-9-2.

To our surprise we identified a series of putative miR-9 response elements in the 3′UTR of HDAC4 mRNAs. Thus, miR-9 has the capacity to reduce the inhibitory activity of HDAC4, stabilizing its own expression in a reinforcing, positive feedback mechanism which enhances the neurogenic capacity of neural precursor cells (Fig. 5). Interestingly, a similar mechanism occurs during myocyte differentiation. MiR-1, a muscle specific miRNA, has been shown to inhibit HDAC4 which in turn de-represses Mef2C, allowing myocyte differentiation to proceed [62]. This form of miRNA regulation supports the notion that miRNAs serve to canalize cellular differentiation. During neural precursor cell differentiation, regulated miRNAs are likely to modulate or restrict expression of genes which can repress differentiation or are associated with alternate cellular fates.

The identification of miRNA transcriptional control elements that are regulated during neurogenesis and modulated by the same miRNA targeting a potential inhibitory element demonstrates a powerful scheme for promoting the canalization of neuronal fate. This regulatory pattern also exemplifies the theme that transcription factors, miRNAs, and regulatory factors all interact during the production of mature phenotypes from precursor cells.

## Supporting Information

Figure S1 **Evolutionary expansion of the miR-9 family of microRNAs.** A) Cladogram describing the differences between 78 selected members of the miR-9 gene family (see File S1) across all species present in miRBase v20. B) Mature rat miR-9 can be derived from multiple potential genomic loci, two of which are immediately adjacent to known Mef2-encoding genes. miR-9-1 and miR-9-2 are both located on chromosome 2 in the rat, and are immediately adjacent to two distinct Mef2 gene paralogs. miR-9-3 is relegated to chromosome 1 and does not have a neighboring Mef2 gene, but does however, retain a member of the Rhesus blood group associated family (Rhbg) as seen next to miR-9-1. While no identified Mef2 isoform is near miR-9-3, the region contains numerous ESTs derived from embryonic brain cDNA libraries (not shown). C) Alignment of proposed Mef2 binding site (Mef2.1) from rat genome, with positions of cloning primers shown for deletion analysis, with representative mammalian genome sequences. D) Alignment of proposed Mef2.2 site from rat with mammalian genome sequences.
(TIF)

Figure S2 **Reconstructed mRNA and Protein diagrams for Rat Mef2c.** Two regions of homology to mouse and human Mef2c were combined to produce a new predicted transcript. Since no sequence overlap was available, the two fragments were joined in the appropriate orientation and key features of the new transcript were annotated based on homology to mouse and human. A) Known mouse exon-intron boundaries, as well as estimated 5′ and 3′ untranslated regions were mapped to the rat genomic region. B) The mRNA sequence was translated into a putative Mef2c protein sequence and key residues and domains were identified based on homology to the human Mef2c. C) The resulting full-length protein sequence is presented.
(TIF)

Figure S3 **Identification of predicted Mef2 binding sites upstream of mouse mir-9-2.** The sequence shown is chr13: 83,732,814-83,738,885 (+) from the mm10 genome. The miR-9-2 mature sequence is underlined at the 3′ end of the sequence. Green highlighting identifies sites predicted by Consite/Jaspar to bind Mef2 with a higher score, while yellow identifies predicted sites with a lower score. Regions surrounding predicted sites were extracted and used to create PCR primers for use in Fig. 3C. Predicted mouse and rat Mef2 binding sites, while homologous in sequence (Fig. S1C&D) appear in somewhat different positions relative to the miR-9-2 transcript.
(PDF)

Figure S4 **Construction of HDAC4 response element plasmids.** The response element sequences were designed based on mRNA sequences and constructed using complimentary oligos, as shown below. The complementary oligos were hybridized and then ligated into the pMIR-Luciferase plasmid. The four oligos are indicated with different colors.
(PDF)

Table S1 **PCR primers used in this study.**
(DOCX)

Table S2 **Predicted upstream Mef2 binding sites for a group of brain-enriched microRNAs and a random subset of microRNAs expressed but not regulated in differentiating L2.2 and L2.3 cells.** Mef2 binding sites within a 5 Kb upstream region of isoforms of the brain-enriched microRNAs miR-9 and miR-124 are enriched as compared to a random set of expressed microRNA upstream regions. The prevalence for Mef2 binding sites in these regions is highlighted by the sheer absence of predicted sites in the random subset.
(DOCX)

File S1 **78 select miRNA precursor sequences for members of the miR-9 family (miRBase v20).**
(ZIP)
