# Correction: A Positive Feedback Mechanism That Regulates Expression of miR-9 during Neurogenesis

**DOI:** 10.1371/journal.pone.0101029

**Published:** 2014-06-20

**Authors:** The PLOS ONE Staff

## Notice of Republication

This article was republished on May 23, 2014, to correct Figure 1, which was an accidental duplication of Figure S1. Please download this article again to view the correct version. The originally published, uncorrected article and the republished, corrected article are provided here for reference.

## Supporting Information

File S1 Originally published, uncorrected article.
(PDF)

File S2 Republished, corrected article.
(PDF)
