# Supplementary figures and images for: A Positive Feedback Mechanism That Regulates Expression of miR-9 during Neurogenesis
Source: PLoS One. 2014 Apr 8;9(4):e94348. doi: 10.1371/journal.pone.0094348 (PMC3979806; doi:10.1371/journal.pone.0094348)

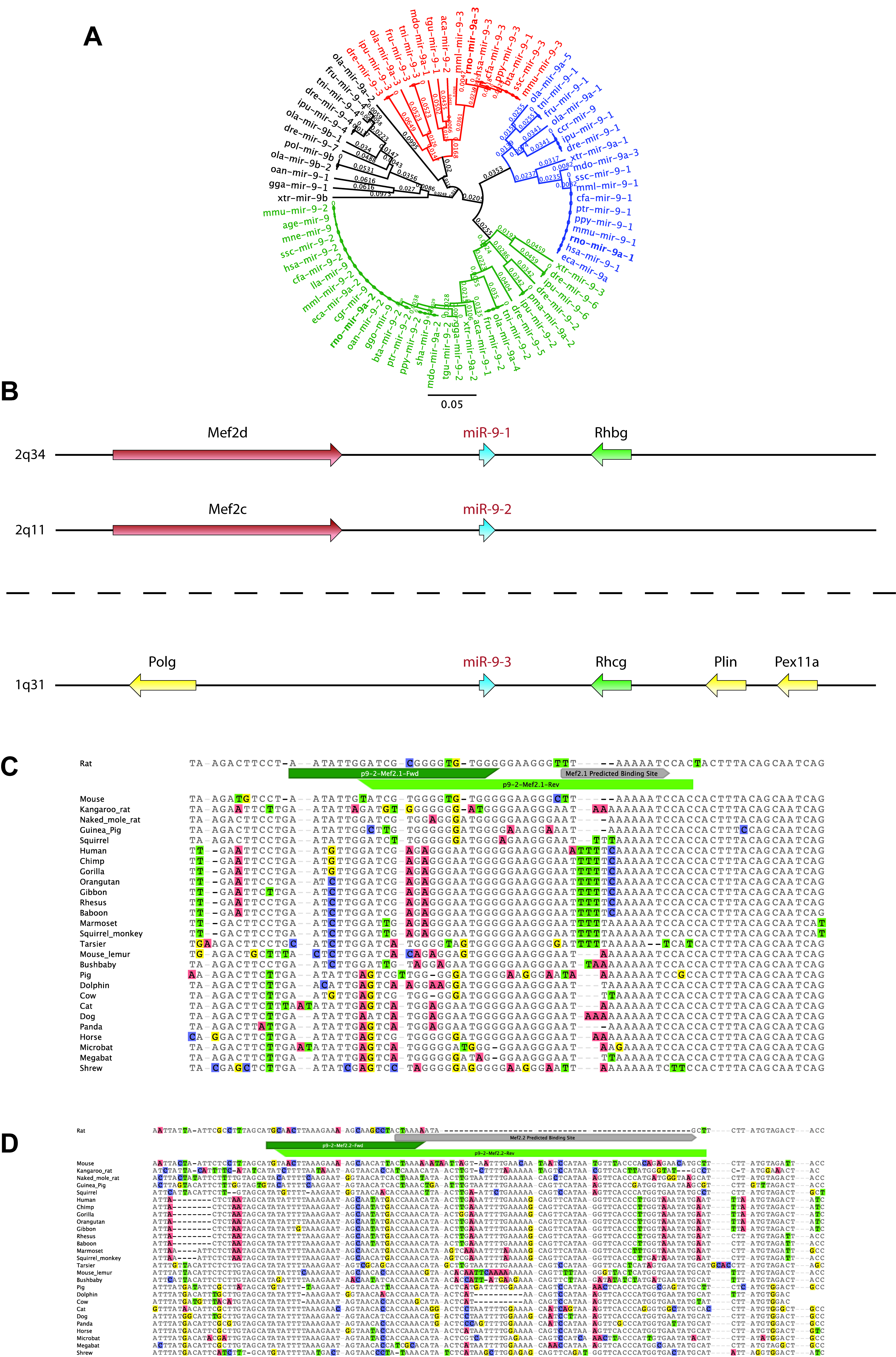

Supplement: Figure S1 — Evolutionary expansion of the miR-9 family of microRNAs. A) Cladogram describing the differences between 78 selected members of the miR-9 gene family (see File S1) across all species present in miRBase v20. B) Mature rat miR-9 can be derived from multiple potential genomic loci, two of which are immediately adjacent to known Mef2-encoding genes. miR-9-1 and miR-9-2 are both located on chromosome 2 in the rat, and are immediately adjacent to two distinct Mef2 gene paralogs. miR-9-3 is relegated to chromosome 1 and does not have a neighboring Mef2 gene, but does however, retain a member of the Rhesus blood group associated family (Rhbg) as seen next to miR-9-1. While no identified Mef2 isoform is near miR-9-3, the region contains numerous ESTs derived from embryonic brain cDNA libraries (not shown). C) Alignment of proposed Mef2 binding site (Mef2.1) from rat genome, with positions of cloning primers shown for deletion analysis, with representative mammalian genome sequences. D) Alignment of proposed Mef2.2 site from rat with mammalian genome sequences. (TIF) [file pone.0094348.s001.tif]
