# Supplementary material for: A Positive Feedback Mechanism That Regulates Expression of miR-9 during Neurogenesis
Source: PLoS One. 2014 Apr 8;9(4):e94348. doi: 10.1371/journal.pone.0094348 (PMC3979806; doi:10.1371/journal.pone.0094348)
Supplement: Figure S3 — Identification of predicted Mef2 binding sites upstream of mouse mir-9-2. The sequence shown is chr13: 83,732,814-83,738,885 (+) from the mm10 genome. The miR-9-2 mature sequence is underlined at the 3′ end of the sequence. Green highlighting identifies sites predicted by Consite/Jaspar to bind Mef2 with a higher score, while yellow identifies predicted sites with a lower score. Regions surrounding predicted sites were extracted and used to create PCR primers for use in Fig. 3C. Predicted mouse and rat Mef2 binding sites, while homologous in sequence (Fig. S1C&D) appear in somewhat different positions relative to the miR-9-2 transcript. (PDF) [file pone.0094348.s003.pdf]

Figure S3. Identification of predicted Mef2 binding sites upstream of mouse mir-9-2. The sequence shown is chr13: 83,732,814-83,738,885 (+) from the mm10 genome. The miR-9-2 mature sequence is underlined at the 3' end of the sequence. Green highlighting identifies sites predicted by Consite/Jaspar to bind Mef2 with a higher score, while yellow identifies predicted sites with a lower score. Regions surrounding predicted sites were extracted and used to create PCR primers for use in Fig. 3C. Predicted mouse and rat Mef2 binding sites, while homologous in sequence (Fig. S1C&D) appear in somewhat different positions relative to the miR-9-2 transcript.

CCTGGCGCAGACAGATCAAGTGAGTATCTTGGCGGCATGAAGGCTGCATG
CTTCCCCGGTTACGCCAGGCCCGCAGCTCCCGTGGTTTGTCCGGCACCCA
CACAAGGGCCTGGCGAGGGCCGCTCCGAGGGCCAGTCCGGGGCTTTGCTT
CAGCTAATTGATTGTATGTCTCCAATACCAAACCTGTTGAACTCGTGAGT
TGTCCGAGCCAGGACTGTTAATTATCCTTTGGGCAGACCCCTAATTTTAG
AAAGGTGCCAGGAAAATTCGCACAGGGAAAAGAAAAAGGACGTAAATTAA
TAGTATTGGTGGAGAGACTTTCTTCTCTTAGATGCCGTGCTAAGTGAAAT
TGGGTTTTGCGTTTTTGAATTCTGTATTCTCATTTTGAATGTGCTGTTTT
TGAATAGCCCCCAAAGTTTCAGTTACCTGGCACTGAAGTCACGGTGGAAA
ACTTACGTAAAATAGCTCGTTGCATCTGGTAGATTCTCCTCCCCCCACAC
CCTTTTTTCTTCCACTTTTTGGGTTTTGGAGGTGAGAGACTGCTTTATAG
GGTTACTCCAAGTTTAGCTTTAACCGAGATGCGACCTTCGTGGTTAGTTA
TTGGGACTTGGAGCTATTCCATTTCAGATGCCTCTGGAATCGGAGCGAAG
CGAGTTCTCTTGCTTAAGAGCCAACCAACTGAAGGCAAGATCTCTTTCGG
TGGTTAGGAACTATCTGCAGAAAATCCTGAGCAAAGAGTACCTAGCCCCC
CCCCCCCCGCCCCCATTAGGATCTGCCGCTTTTTTCTCTTGCAAATGTGC
AGGCGGTGTTTGAATAGAATACTGTCTGGAGGGGTCAGGGCAATTCAGTG
GAATTGATCAGAAGTTAGACCCCAGGCCCTGTCATGTCACGGCCACGGCC
GTGGGGTGGGGGTGGGGGTTAAAGCTGTGGCTGTCTTGGGTCCACAACTA
TGGAGTCAGATCGTTTTCTCTCTCTCCTGGCGCCCACACCTGCCCACCAC
CCTTCATCTCCACCTTGGCACCAGTAACGAGGGCGCTGTTAGGAACACGC
CACAGTAGTCAGCATAATTGAACCGGTGAATACTTTCATTTTGCAAAACA
ACAGCTCCACTCCTCCAGTCCAAAATAATAGTAAGCATCAGAAAATCATT
TTTAAAACACGCAACCTATTTTCGGGAGTATGTAAGATGTCCTAATATTG
TATCGTGGGGTGTGGGGGAAGGG**C**TTAAAAATCCAC**C**ACTTTACAGCAAT
CAGGTTTTACATTATTCATTGATTTACTGAAGGAAAGAGCCAATCAGGAG
CCATAGATATGAATGCACATTTCACTGTGCTGGAGCCTTACAGTAAACTA
TTTGCATCTTTCTCATGGATTAGACATTTATCCTGGGATTCCCATTGTTT
GAAAGGCCAAGAAATTCAGTCTCTTAAACATTTAGAAAACAAGACTTTGT
TCCAGACTTAAAATCCTTGCATGGTGGGGCTCTCTCTTCTCTTTTGTATG
GGAGGTTACAAGGTTCAAATCCCTAATGTTTCTTAAGTCTCTCTTCTTTC
CAAGCTAAAAATAAAAGGAGAAAGAAAAATTAATTTCCCAAACCCCTCTA
GATCCTGCATATTTTGCATTCCCATTATCATGCATGTCTCCATTTAAATG
CAAATTAGCATATTTAACCCTGTAGGTGCTAAGGCCCAAGATAGACAGTA
CTGGTGTTTTATATAATCTATTGGCATTAAAAAAAAAAAAAACACAGTAAT
AGTGACCCTTTAAATTAAGTCATATTCCTATATAATGATATATATATATA
CATATATATATATACTGTGATCATTTGTTTTATTGTCTGTTTTTCAGATG
ACACTTCAAAGAAGCATAAAATGAACAGACTATTCAAACAAAGGTAATTT
ACATTTTTTTATTTGATAAAACCTGACTGATGTACAAAACGAAAATAGAA
TATTGTTTACTGGAAAACAGGTCTACAATCTCTCACACATCTTCTCTTGC
AACAGATAGAATTCCAGAAACATCTAAGTTCCTCTGTGTACAGTTATTTC
TCCTGTAGGAGCCTTTGTATGGATGTAATACACACATATATGTATATGCA
TAGAGCATGACATAAATAAAACCTGCAATAGCCTATAAGAAAGAAGCTCC
TATTTAGAGGAAAGGGTAAGATACTTTTATTATAATGAAAGCCAGTTCAC
TTCATCACTTGGACCATGGACACTAATGTAAATAAAATGGCTTAAAAAAT
AAGACAATAGTAGTATTGACTGTAGACTACAATTTGCACATTTGTAATTC
TTATAAATGAAATATTTAAAATGATTTGGTGTTGGGCCAGTTCCTTATGC
ATTCTGCATTCATGTGTATAGGATTCAAAGACGTTCCAAATATTGATCAT

AAACCAAGTGTATATGAATTAATTAAATAGCATATTTCTGGCCCAAATAT
GAGGAAAACCATAACTTTTTACTGTAAATCAGGAGAGATTGTTTTATCCC
CAAATAAACTAGGATGTTTCAAGTCATGTTTCAGCAATTTGGAGACCTAC
AAACTATCTGCAAGCAAGCACAACAACCATACTCTAATCTACTAGATAGT
AGTGAGAATTTAGTGCTTGAATTCAGGCTGCTTGAATTCAGTATATCCTT
CTCTATGCTGATTTTCAAGTAGATATTCTGTAGCTCAGCTAGATAATCTT
TTAAAAGCAAAAAAAAAATTAAGTTTAAAGTGTAGTTGCTAAGAGCTGCT
CTTCACAGGTGACATCGCTAGGAGTCTTTGAAGATATGTAATTGTAACGT
ATTGTGGGGTATATATAATACATACAATAAACCATTTTATAAACCTTGGC
AATCTTATAAATAAGGGGGCACTCGATGTTAAATCTTAGTATATTTAATT
TCTTCTGTGTGCCTGTGAGGTTGATAGAGAGTTAAAATATTTAATTGACT
ACATTGCTAATGAACAGACTACCCATAATCCTGATCTTGGCTCATTTCAC
TCATTGGAGTTCCCATGATGAATACCAAGCGTGGAGACCGGCCTTGTAGT
AAGTTGGCATGACTAGTGTTGCCCTAAGAATGTGGTATTTATTACAGCAT
CTTATTCAGATTCTAGTGGGAGAAGCGTTCAGATATGCAGTGTGTTAACT
CATTGTAGATATTGTTTCTAAAAACACATTTTATTGTTAGGTATGGGAAT
AGAGTCATAATAACTTTCAAATTCAGAAAATATGTGAATAAATAACACCC
TCCCAACACATAGATATATATATGTAGCTAGCACACCTAGATACATGTGA
AGAAAAGAGATTCTATAAGACCATTTTTGATTTCTTGGGCTTTGGCTTAC
ACAGTTGACTATATCTGATCACGTGCTTATTTTATGTAATACAAGCCAAA
TCATTAATTACTCTGCTGCTAGTTCTTGGAAAGCAAGGCCACGGTGCTCT
TTAATCTTCCAAGCTGCAAAGCTCATTAGGATTTTTACATGATGTCAGAA
GTGGCATGCGCTTCACTGAGTTGTTTATGCTGTGACCATATGGTTTAGGA
ATGAACAAAAACTCTATGAACATGCCTTTAGGATTGGATTGTAGTGTCCG
TGTGTATTTTATTTCTGACTTCTCGTTCATAATTTGAAGAGAAATATAGA
CGAACTCTTTCCACATCTTAGTCTTTTCTCAATAGCCAGTTACAGAATGC
TTACCATAAAAGTAAACAAATTAATATAAGGACAAATTGGCATCTCTGTT
ACTAAGCTGTAAGCCATATCTTAATTGGTGTGATATGTTTAAACTCTTCA
TTGGAGATGTTTCCTATCATTAAAACACAAACCAAACAAACAAACAAAAA
AAACTTGTCAGTCTCTTCTTTACAGAGTTTAGATTTTAGGGCTCTGTATG
TGATTAAATAACCAGGTTCTTATTTATGTGTTAAATGAATAAATTAGTGA
ATTACTGAGAAAAATTTTATTCCAAAAAAATTAATAAATCCAAAAGTATT
TTAGCCACTGGGGAAAATAGACACATTATGGAATTGAGAGCATTGTGAGG
CACAGGTGATGTGTGCAATGTACATTTAGAATTACTAATTCTCCTTTAGC
ATG**T**AACTTAAAGAAAAGCAA**CAT**TACTAAAAAAATAATTAGTAATTTGAA
CAATAATCCATAATGTTTACCCACAGAGAACATGCTTCTTATGTAGA**T**TA
CCATGAATATTTTGTTGAGAGGGATTTGAATGTTAGATATTAGATGTGCT
AGACAGATTATCTATTATAAAGGATGAGAAAGAAAAGGATTCTTTGAAGT
GATTTTAAAGTCACACTTTAAAAACTCCTAAGACAATGAATGAAGCTAAT
TAATTAATGAAATGACCTTCTGCTTCTCGATAGAAGGATATGAGTGGGTG
GGGCCAGGGAGGTGTGGTAGCTTCCCTCCCTCCCCATCCAGGTGTTGGAT
CTACATGTTCAGGAGACAAGACTTTTATTTTAATTTTATAAGCAAGTCTA
GTGCATTATTGTCCAGTGTGAAATTTATGTTAAAACAGTAGACAAAGGCT
CAGCATTTTTGCTACAGCAGAGTTGTGTACAACATTGAAACTATTGCTGC
AATGTTTACCTGTGCAGCATGCTAACCCCCACACACACTCTATATGCAAA
GCATTTTTAAAAAAGAAGCCCCACTGTTTAGATCGTGCAGTTCGAACTTA
CAGAACAAATATGCTGCCCTATCGCCACGGTACACAGATAGAAAACATGA
CTGAGCTGTAAAGCCATTAAAAACATAACACCAAAGCAAATTGTCATTGT
AACCATCGACTCACTGCGTAAAAACAGAGCTGATTCCCAGCCCCAGAAAG
CGAGGCCCTGCTTTAAGCAATAGTAAAGTGCCTTCCATCTGCCTGCCTAT
GCAATCTGCCAGAAGAGAAATGCAGGCAAAGAAGTTCCCCAGACCTGAAA
TGCTTTTATCTATCCTTAGAGAGAGATTTTCTCCATTCTTTGGATATTAG
AGCGATCTACACTAGCGCCAAGGAGGTAAAAGGCCGCGGGTTGCTTTTCA
AGGAGCAGTCCGTAGTGCTAGTGGATGAAATGTTGATTGAGAATATAGGG
ACTGCTGCAAAGCTGCTGCTGCCGTCGCCGCCACCGCTGCCGCCGTCGCC
GCCGCCGTCGCCTGTGCTACTCCTGGCAGCAGGCGTGGGGCAGTTGGGTG
ACCACTGATGCATATCAATAACCATGTACTTGAGATTTCTCATGACATCA

TCATTACCTTGGTCTCCCGCAGTCTCAAACCTTTGACCTTGCCGGGCTGG
TCCACAATTTGGCGGTGAAAGCCCCTCCAATGATGGCCCTTTTCTCCTTC
TTTCAGACCGGGGCAGCAGTACGCCAGCAAGAGGGCATGCTCTGCCCGCC
TAGAAGTCCTAAGAAATCGAGGATCTCCCTTAAAACATTCACATACAAGA
TATGTGCAATCAAGCTGCCTGCACTTGGTAAAAAAGATCATGATGCCTAA
CCCAATGATCCGGAGCAGATCTTTGAATCAGCTGTCACTTCTATAGAATG
GGATTTCCCCCTTCTTCCTCTTTAACCAGCGTTGGAAACTGCCGATGGCT
TTATACATCTCAGTAGAGGAAGGCTAAAGAATCTTCCGTGAGAATGAAGC
AATTTACAATGCAAATAGAGGCCTGTGAAGAGAAGGACACTGCTGCTCAC
CCCCAACAGCTTGCTGCACCTTAGTCTCTGGTCTTAATTTCTGATACTCA
TAGATGAATTGTTGGCTAACGATACTGGAGATTACTTGCTGGAAGGGGAA
AAGTTTGGAATAAACTCTGAATGCAACAGATCTCATTTGGATGTTCTAGC
CGCACACACTAAGACAGAAACCAGGTCATGTTTACATGAAAGGAGAAGCT
TAAACGCGGCAAGTACCCCGGAGGACTACGCTTCCCTAAAGCCAAAGAGG
ATCGAGATCTGGAGTTCAGCCAGAGGAAGGCAGCCTTGTGAGGGAAGCGA
GTTGTTATCTTTGGTTATCTAGCTGTATGAGTGTATTGGTCTTCATAAAG
CTAGATAACCGAAAGTAAAAAC
