# Supplementary material for: A Positive Feedback Mechanism That Regulates Expression of miR-9 during Neurogenesis
Source: PLoS One. 2014 Apr 8;9(4):e94348. doi: 10.1371/journal.pone.0094348 (PMC3979806; doi:10.1371/journal.pone.0094348)
Supplement: Figure S4 — Construction of HDAC4 response element plasmids. The response element sequences were designed based on mRNA sequences and constructed using complimentary oligos, as shown below. The complementary oligos were hybridized and then ligated into the pMIR-Luciferase plasmid. The four oligos are indicated with different colors. (PDF) [file pone.0094348.s004.pdf]

Figure S4. Construction of HDAC4 response element plasmids. The response element sequences were designed based on mRNA sequences and constructed using complimentary oligos, as shown below. The complementary oligos were hybridized and then ligated into the pMIR-Luciferase plasmid. The four oligos are indicated with different colors.

Oligos for HDAC4 Response Elements

RE1

```
HindIII                                                           SpeI
+agctgcggcggcggcgttcgctgcttttggaactacctcaccttccttaaacggtgatcaaac
    -cgccgccgccgcaagcgacgaaaaaccttgatggagtggaaggaatttgccactagtttggatc
```

RE1mt

```
HindIII                                                           SpeI
+agctgcggcggcggcgttcgctgctaaaaccaactacctcaccttccttaaacggtgatcaaac
    -cgccgccgccgcaagcgacgattttggttgatggagtggaaggaatttgccactagtttggatc
```

Oligo	Sequence (5’ to 3’)
HDAC4 RE1.1	agctgcggcggcggcgttcgctgcttttgg
HDAC4 RE1.2	aactacctcaccttccttaaacggtgatcaaac
HDAC4 RE1.3	aggtagttccaaaaagcagcgaacgccgccgccgc
HDAC4 RE1.4	ctaggtttgatcaccgtttaaggaaggtg
HDAC4 RE1.1 mt	agctgcggcggcggcgttcgctgctaaaacc
HDAC4 RE1.2	aactacctcaccttccttaaacggtgatcaaac
HDAC4 RE1.3 mt	aggtagttggttttagcagcgaacgccgccgccgc
HDAC4 RE1.4	ctaggtttgatcaccgtttaaggaaggtg
