# Supplementary material for: A Positive Feedback Mechanism That Regulates Expression of miR-9 during Neurogenesis
Source: PLoS One. 2014 Apr 8;9(4):e94348. doi: 10.1371/journal.pone.0094348 (PMC3979806; doi:10.1371/journal.pone.0094348)
Supplement: Table S1 — PCR primers used in this study. (DOCX) [file pone.0094348.s005.docx]

Table S1. PCR primers used in this study.

| **Mef2 Family mRNA qPCR Primers** | | |
| --- | --- | --- |
| **Primer** | **Species** | **Sequence** |
| SP-Mef2a Rat RT | rat | TCGGAAACCAGATCTACGGG |
| ASP-Mef2a Rat RT | rat | ATCATGCCCTTGCTTGAAGG |
| SP-Mef2b Rat RT | rat | CGCCTCTTCCAGTATGCGAG |
| ASP-Mef2b Rat RT | rat | GTATTTGAGCAGCACCCGGT |
| SP-Mef2c Rat P1 | rat | TGCTCAAGTACACCGAGTACAACGAGC |
| ASP-Mef2c Rat P2 | rat | CAGACCACCTGTGTTACCTGCACTTGG |
| SP-Mef2d Rat | rat | ATCGCGCTCATCATCTTCAA |
| ASP-Mef2d Rat | rat | GCTGGCGTACTGAAACAGCTT |
|  |  |  |
| **rno-miR-9 Primary transcript/precursor qPCR Primers** | | |
| **Primer** | **Species** | **Sequence** |
| SP/pre-rno-mir-9-1/100-200 | rat | TGGTGTGGAGTCTTCATAAAGCT |
| ASP/pre-rno-mir-9-1/100-200 | rat | ACCAACGTTCCAGGCCCTCT |
| SP/pre-rno-mir-9-2/111-200 | rat | CTCATACAGCTAGATAACCAAAGAT |
| ASP/pre-rno-mir-9-2/111-200 | rat | GATCCGGATCTGGAGTCCAG |
| SP/pre-rno-mir-9-3/57-146 | rat | CCGTTTCTCTCTTTGGTTAT |
| ASP/pre-rno-mir-9-3/57-146 | rat | TTCGGTTATCTAGCTTTATG |
| SP/pri-mir-9-1 | rat | CGCTAAAGCCATCCCAGGA |
| ASP/pri-mir-9-1 | rat | TCCCAACATAGCCCACTGCT |
| SP/pri-mir-9-2 | rat | GCATTTCCCACCCACAGC |
| ASP/pri-mir-9-2 | rat | CAGTTGGTGTTGCCATTTGG |
| SP/pri-mir-9-3 | rat | CCCCATACTGCATTGTCCCT |
| ASP/pri-mir-9-3 | rat | ACACGCTCGCTTCTGGATTT |
|  |  |  |
| **Mouse Mef2 Binding Site qPCR Primers** | | |
| **Primer** | **Species** | **Sequence** |
| Mef2-1-mir9-2-fwd | mouse | TCCGAGCCAGGACTGTTAAT |
| Mef2-1-mir9-2-rev | mouse | TTTCACTTAGCACGGCATCT |
| Mef2-2-mir9-2-fwd | mouse | CTCCACTCCTCCAGTCCAAA |
| Mef2-2-mir9-2-rev | mouse | CCTGATTGCTGTAAAGTGGTG |
| Mef2-3-mir9-2-fwd | mouse | GGTGGGGCTCTCTCTTCTCT |
| Mef2-3-mir9-2-rev | mouse | GCATGATAATGGGAATGCAA |
