# Supplementary material for: A Positive Feedback Mechanism That Regulates Expression of miR-9 during Neurogenesis
Source: PLoS One. 2014 Apr 8;9(4):e94348. doi: 10.1371/journal.pone.0094348 (PMC3979806; doi:10.1371/journal.pone.0094348)
Supplement: Table S2 — Predicted upstream Mef2 binding sites for a group of brain-enriched microRNAs and a random subset of microRNAs expressed but not regulated in differentiating L2.2 and L2.3 cells. Mef2 binding sites within a 5 Kb upstream region of isoforms of the brain-enriched microRNAs miR-9 and miR-124 are enriched as compared to a random set of expressed microRNA upstream regions. The prevalence for Mef2 binding sites in these regions is highlighted by the sheer absence of predicted sites in the random subset. (DOCX) [file pone.0094348.s006.docx]

Table S2. Predicted upstream Mef2 binding sites for a group of brain-enriched microRNAs and a random subset of microRNAs expressed but not regulated in differentiating L2.2 and L2.3 cells. Mef2 binding sites within a 5Kb upstream region of isoforms of the brain-enriched microRNAs miR-9 and miR-124 are enriched as compared to a random set of expressed microRNA upstream regions. The prevalence for Mef2 binding sites in these regions is highlighted by the sheer absence of predicted sites in the random subset.

| ***Upstream region*** | ***Predicted Mef2 binding sites*** | ***100% Core match to PWM*** | ***Matrix Match > 90%*** |
| --- | --- | --- | --- |
| miR-9-1 | 1 | 1 | 1 |
| miR-9-2 | 3 | 2 | 2 |
| miR-9-3 | 1 | 0 | 0 |
| miR-124a-1 | 1 | 1 | 0 |
| miR-124a-2 | 2 | 2 | 1 |
| miR-124a-3 | 0 | 0 | 0 |
| miR-190 | 0 | 0 | 0 |
| miR-34b | 0 | 0 | 0 |
| miR-142 | 0 | 0 | 0 |
| miR-130b | 0 | 0 | 0 |
| miR-339 | 0 | 0 | 0 |
| miR-25 | 0 | 0 | 0 |
| miR-93 | 0 | 0 | 0 |
| ***P-value*** | ***0.017225*** | ***0.040859*** | ***0.101939*** |
